# Coronavirus in human diseases: Mechanisms and advances in clinical treatment

**DOI:** 10.1002/mco2.26

**Published:** 2020-10-01

**Authors:** Panpan Lin, Manni Wang, Yuquan Wei, Taewan Kim, Xiawei Wei

**Affiliations:** ^1^ Laboratory of Aging Research and Cancer Drug Target State Key Laboratory of Biotherapy and Cancer Center National Clinical Research Center for Geriatrics West China Hospital Sichuan University Chengdu China; ^2^ Wexner Medical Center The Ohio State University Columbus Ohio 43210 USA

**Keywords:** clinical treatments, COVID‐19, human coronaviruses, MERS, SARS

## Abstract

Coronaviruses (CoVs), a subfamily of coronavirinae, are a panel of single‐stranded RNA virus. Human coronavirus (HCoV) strains (HCoV‐229E, HCoV‐OC43, HCoV‐HKU1, HCoV‐NL63) usually cause mild upper respiratory diseases and are believed to be harmless. However, other HCoVs, associated with severe acute respiratory syndrome, Middle East respiratory syndrome, and COVID‐19, have been identified as important pathogens due to their potent infectivity and lethality worldwide. Moreover, currently, no effective antiviral drugs treatments are available so far. In this review, we summarize the biological characters of HCoVs, their association with human diseases, and current therapeutic options for the three severe HCoVs. We also highlight the discussion about novel treatment strategies for HCoVs infections.

## Graphical abstract

Current mechanisms of anti‐CoV therapeutic strategies. The idea to disturb the normal life cycle of the virus provides significant insights into the clinical treatment strategies. All of SARS‐CoV, MERS‐CoV, and SARS‐CoV‐2 encode structure proteins (like S protein), nonstructure proteins (eg, PLpro, 3CLpro, RdRp, and helicase), and accessory proteins that are essential for the viral life cycle and that are considered as important targets for the development of antiviral agents. Additionally, enhancement of INF response and several other cell signaling pathways are also regarded as potential anti‐CoV strategies.

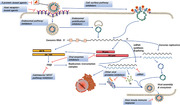

## 1 INTRODUCTION

Coronaviruses (CoVs) is a family of enveloped, positive, single‐stranded RNA viruses, which are infectious to animals and people, and are able to cause respiratory, hepatic, enteric, and neurological diseases of various severity.^1^, ^2^ Based on their genetic relationship and genomic structures, this family is divided into four genera, termed Alpha‐CoV, Beta‐CoV, Gamma‐CoV, and Delta‐CoV.^3^, ^4^, ^5^ Among the seven identified human coronaviruses, HCoV‐NL63 and HCoV‐229E belong to the Alpha‐CoV, and the other five types are classified as Beta‐CoV, including HCoV‐OC43, HCoV‐HKU1, SARS‐CoV, MERS‐CoV, and SARS‐CoV‐2.^6^

It was not until the outbreak of severe acute respiratory syndrome (SARS) in 2002/2003 and Middle East respiratory syndrome (MERS) in 2012 that CoVs were considered as a fatal threat to human beings and received global attention,^7^, ^8^ although they have been discovered for decades.^9^, ^10^ In addition to SARS and MERS, other human CoVs generally cause only mild upper respiratory diseases, which is similar to common flu.^11^, ^12^ A novel CoV, named SARS‐CoV‐2 by the World Health Organization (WHO), has emerged again at the end of 2019, causing more infections and deaths worldwide than ever before.^13^ The absence of effective antiviral treatments and serious consequences of these three CoVs have highlighted the urgent need for novel drug development to prevent the spread of CoVs.

Herein, this review mainly focuses on the biological characters of HCoVs, their association with human diseases, and current therapeutic options for the three severe HCoVs. We also highlight the discussion about novel treatment strategies for HCoVs infections.

## 2 BIOLOGICAL CHARACTERS OF CORONAVIRUSES

### 2.1 Genomes

**FIGURE 1:**
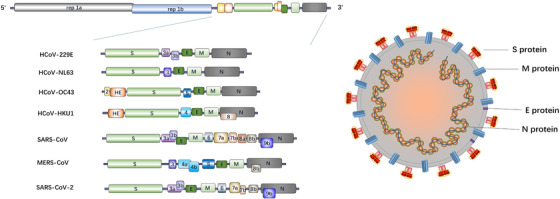
Genome organization and structure of HCoVs

CoVs possess a nonsegmented, positive, single‐stranded RNA genome of 26‐32 kb.^2^, ^14^, ^15^ All CoVs have a similar genome arrangement with a 5′‐methylated cap structure along with 3′‐polyadenylated tail. The replicase gene, occupying about 20 kb, two‐thirds of the genome and comprising two open reading frames (ORFs), ORF1a and ORF1b, is located at the 5′ end.^2^ It encodes two large polyproteins (pp) 1a and 1ab that can be cleaved by papain‐like cysteine protease (PLpro) and 3C‐like serine protease (3CLpro) into nonstructure proteins, involving some proteases, several RNA modification enzymes, as well as RNA‐dependent RNA polymerase (RdRp) and helicase (Hel) required for virus replication.^16^ Additionally, an untranslated region (UTR) can also be identified at the 5′‐end as same as the 3′‐terminal. Structure proteins, encompassing the 3′‐terminal one‐third of the genome, are arranged in a certain order of hemagglutinin esterase (HE) protein that is present in some beta‐CoVs, spike protein (S), small membrane protein (E), membrane protein (M), and nucleocapsid protein (N). in brief, the arrangement of the CoV genome can be shown as 5′‐UTR‐replicase gene (ORF 1a and ORF 1b)‐HE protein (if have)‐S protein‐E protein‐M protein‐N protein‐3′ UTR‐poly (A)^2^ (Figure 1).

### 2.2 Virion structures

CoV is named for the club‐shaped projections eradiating from the envelope, which forms its corona or crow‐like morphology. The shape of the viral particles is roughly spherical with approximately 80–160 nm in diameters.^17^, ^18^, ^19^ The nucleocapsid protein and the genome RNA intertwine to form a helical structure located inside the envelope. For some CoVs, the spikes on the surface are not only formed by trimers of S protein, but also HE proteins. M protein and E protein, two transmembrane proteins, also participate in the composition of the virus (Figure 1).

S protein, a transmembrane protein, mediates the initiation of CoV infection by attaching to the specific receptors on the target cells.^20^, ^21^, ^22^ For a prototypical CoV, S protein is usually cleaved into an extracellular receptor binding subunit (S1) and a membrane‐anchored subunit (S2), responsible for virus binding and membrane fusion, respectively.^23^, ^24^ Two heptad repeats (HR1 and HR2) and enriched alpha‐helices are contained in the S2 domain, a feature typical of fusion protein.^25^, ^26^, ^27^ The receptor‐binding domain (RBD) of S protein specifically binds to target receptors, leading to the conformation change of S1/S2 complex that mediates virus entry.^26^ Furthermore, the RBD also induces potent neutralizing Ab response, which turns S protein into an important antigenic determinant capable of protective immunity induction and provides a vital approach for the development of immunotherapies.^28^, ^29^, ^30^, ^31^, ^32^

CoV E protein (8.4‐12 kDa) is an integral membrane protein of 76–109 amino acids^33^, ^34^, ^35^ and is present in small amount in virions.^36^, ^37^, ^38^ It contains a short hydrophilic N‐terminal followed by a single predicted hydrophobic domain and a hydrophilic C‐terminal. Although its membrane topology remains unclear, CoV E protein is commonly referred to as a transmembrane protein with one transmembrane domain.^39^, ^40^, ^41^ Accordingly, the E protein mainly targets ER and Golgi‐complex and participates in part of the life cycle of the virus, including virus assembly, budding, particles release, envelope formation, and viral pathogenesis.^33^, ^35^, ^41^, ^42^, ^43^, ^44^, ^45^, ^46^, ^47^, ^48^, ^49^, ^50^ In addition, CoV E protein may have a crucial role in virus production and maturation, because recombinant CoVs lacking the E protein exhibit significantly reduced viral titers and toxicity.^51^, ^52^, ^53^, ^54^, ^55^

The M protein is a small transmembrane protein (25–30 kDa) with three transmembrane segments, an N‐terminal ectodomain and a C terminal‐endodomain, determining the shape of the virion.^56^, ^57^ It is considered as the most abundant structural protein and plays a pivotal role in virion assembly via interacting with other structural proteins.^58^, ^59^, ^60^ Binding of S protein and M protein is essential to the virus assembly and the maintenance of S protein in the ER‐Golgi intermediate compartment (ERGIC)/Golgi complex.^61^, ^62^, ^63^ Virus‐like particles (VLPs) are assembled when the combination of M and E proteins occurs, which suggests that they are required for the formation of the envelope.^39^, ^64^, ^65^, ^66^ Additionally, when expressed alone, it can become a homomultimeric complex, the primary driving force of envelope formation.^43^, ^60^, ^67^ Furthermore, as to N protein, a stable nucleocapsid and internal core of virions can be achieved when combined with M protein.^68^, ^69^

The N protein, combined with viral genomic RNA to form a helical nucleocapsid inside the viral envelope, is a multifunctional protein.^70^ It contains three highly conserved domains: the N‐terminal domain (NTD), responsible for RNA binding; a C‐terminal domain (CTD) that is a hydrophobic and helix‐rich terminal, capable of dimerization and oligomerization; and an intrinsically disordered region (RNA‐binding domain/domain 2) that is a serine/arginine‐rich domain (SR‐domain) with a significant phosphorylation potential.^15^, ^71^, ^72^, ^73^, ^74^, ^75^, ^76^, ^77^, ^78^, ^79^, ^80^, ^81^, ^82^, ^83^, ^84^, ^85^, ^86^ Phosphorylation of the N protein can initiate structural modification leading to an increased RNA‐binding affinity.^72^, ^79^, ^87^, ^88^ The N protein binds to the genomic RNA through a beads‐on‐a‐string form. Likewise, except for the interaction between N protein and nucleic, the ability of complex oligomerization is another pivotal activity required for the formation of the ribonucleoprotein complexes for viral assembly.^89^ In addition to the role of the N protein in viral core formation and assembly,^69^, ^90^, ^91^, ^92^ it is also involved in other critical processes of viral life cycle such as virus budding and envelope formation,^21^, ^93^, ^94^, ^95^ genomic mRNA replication ,and genomic RNA synthesis.^96^, ^97^

## 3 PATHOGENESIS OF CORONAVIRUS IN RESPIRATORY DISEASES

Although the pathogenic mechanisms of human CoVs have not yet been fully understood, the investigation of their unique characteristics of each CoV enables to distinguish the various human CoVs including SARS, MERS, and SARS‐COV‐2.

### 3.1 Target receptors and virus entry

#### 3.1.1 Target receptors

The crucial early step of the infection of human CoVs into susceptible host cells is the interaction between viral S protein and cellular target receptors. One of the subunits of S protein, S1, containing RBD, is responsible for specific recognition and binding of the target receptors. The other subunit, S2, is in charge of the membrane fusion.^22^, ^98^, ^99^, ^100^, ^101^, ^102^ The tissue tropism, as well as the susceptible host species, is mainly determined by the binding of S protein to target receptors.^102^ Based on lines of known evidence, human CoVs utilize multiple and different types of cellular receptors rather than use a common receptor. Therefore, multiple cellular receptors have been identified as target receptors for the various human CoVs to date (Table 1).

**TABLE 1:** Biological characteristic of human coronaviruses

	HCoV‐229E	HCoV‐NL63	HCoV‐OC43	HCoV‐HKU1	SARS‐CoV	MERS‐CoV	SARS‐CoV‐2
**Genus**	Alpha‐CoVs	Alpha‐CoVs	Beta‐CoVs	Beta‐CoVs	Beta‐CoVs	Beta‐CoVs	Beta‐CoVs
**Natural reservoir**	Unknown	Unknown	Unknown	Unknown	Bats	Bats	Bats
**Intermediary host**	Unknown	Unknown	Unknown	Unknown	Palm civet	Dromedary camel	Pangolin
**Target receptor**	APN	ACE2	9‐O‐acetylated sialic acid	9‐O‐acetylated sialic acid	ACE2	DPP4	ACE2
**Receptor distribution**	Epithelial cells, endothelial cells, leukocyte, and fibroblast	Heart and vascular endothelia, small intestine epithelia, alveolar macrophage and monocytes, epithelial cells of trachea, bronchi and alveoli	Submaxillary mucin	Submaxillary mucin	Heart and vascular endothelia, small‐intestine epithelia, alveolar macrophage and monocytes, epithelial cells of trachea, bronchi and alveoli	Endothelia cells, epithelial cells, inflammatory cells in lung, and smooth muscle cells	Heart and vascular endothelia, small‐intestine epithelia, alveolar macrophage and monocytes, epithelial cells of trachea, bronchi and alveoli
**Syndromes**	Mild upper respiratory diseases, similar to common flu	Mild upper respiratory diseases, similar to common flu	Mild upper respiratory diseases, similar to common flu	Mild upper respiratory diseases, similar to common flu	SARS syndromes	MERS syndromes	COVID‐19

Abbreviations: ACE2, angiotensin‐converting enzyme 2; APN, aminopeptidase N; CoVs, coronaviruses; DPP4, dipeptidyl peptidase IV; MERS, Middle East respiratory syndrome; SARS, severe acute respiratory syndrome.

Angiotensin‐converting enzyme 2 (ACE2), the first known human homolog of Angiotensin‐converting enzyme and the receptor for HCoV‐NL63, SARS‐CoV, and SARS‐CoV‐2, is a vital component of the renin‐angiotensin system (RAS).^103^, ^104^, ^105^, ^106^, ^107^, ^108^, ^109^ It is a secreted enzyme with a transmembrane domain, a single metalloprotease active site and a signal peptide,^110^ and predominantly expressed in heart, vascular endothelia, epithelia of the small intestine, kidney, and testis; alveolar macrophage and monocytes of the respiratory tract; and epithelial cells of the trachea, bronchi, and alveoli.^103^, ^110^, ^111^, ^112^ In contrast to its homolog ACE that contributes to the promotion of lung failure pathogenesis, induction of lung edemas, and impairment of lung function, ACE2 plays a protective role in severe acute lung injury (ALI). Imai et al revealed that the deficiency of ACE2 in the murine models of acute respiratory distress syndrome (ARDS) deteriorated the symptom in lung function, which could be recovered by the recombinant ACE2.^113^ Thus, downregulation of ACE2 expression in SARS patients could be used as an indicator of severe clinical outcome.^114^ Besides lung damage caused by SARS‐CoV infection could be attenuated by blocking the renin‐angiotensin pathway.^114^ Overall, ACE2 could serve as a novel therapeutic target for severe respiratory diseases.

Dipeptidyl peptidase IV (DPP4), a type II transmembrane protein also known as CD26, is identified as a target receptor for MERS.^115^ It is a prolyl oligopeptidase expressed in various cells including endothelial cells, epithelial cells, and inflammatory cells in the lung and smooth muscle.^116^, ^117^, ^118^ The multifunctional protein DDP4 is implicated in the activation of T‐cell, the activity regulation of chemokines and growth factors, and the regulation of glucose metabolism.^119^, ^120^, ^121^, ^122^ Not only can it be embedded in the plasma membrane in the form of a homodimer, but it also presents in extracellular fluid like plasma as a soluble form.^118^, ^123^ Although DDP4 is expressed in epithelial cells of the upper respiratory tract in camels, it is principally found in alveoli but rarely in the nasal cavity or conducting airway.^115^, ^124^ Accordingly, in a murine MERS model, though monocyte infiltration, alveolar edema and microvascular thrombosis were observed in the MERS‐CoV‐infected lungs, any symptoms were seldom found in the airways.^125^

Aminopeptidase N (APN), a type of II metalloprotease also called CD13, is a ubiquitous enzyme expressed in various organs (lung, intestine, and kidney) and cells (epithelial cells, endothelial cells, leukocyte, and fibroblast).^126^, ^127^ It serves as a target receptor for HCoV‐229E,^128^ but not for HCoV‐OC43. HCoV‐OC43 shares the same specific target with HCoV‐HKU1, namely 9‐O‐acetylated sialic acid.^129^, ^130^

#### 3.1.2 Virus attachment and entry

Virus entry is a finely regulated process requiring a series of interactions between the virion and host cell.^131^, ^132^, ^133^ Following the conjunction with the target receptor, CoV fuse its envelope with the membrane of the host cell. These processes are forced by the conformational change of S protein, which is triggered by not only the target receptor binding but also PH acidification and proteolytic cleavage led by cell surface or endosomal proteases such as transmembrane protease serine 2 (TMPRSS2), furin, cathepsin L, elastase, and trypsin.^134^, ^135^, ^136^, ^137^, ^138^, ^139^, ^140^, ^141^, ^142^, ^143^ Cleavages of S protein are facilitated at two sites: the boundary between the S1 and S2 subunit (S1/S2) and the conserved site upstream of the fusion peptide (S2’).^138^, ^144^ The former one is aimed at releasing RBD from the membrane fusion subunit, and the latter one is important for the exposure of the fusion peptide, hydrophobic in general, which acts as an anchor to target membrane.^138^, ^145^, ^146^ Then the fusion domain adopts two heptad repeats (HR1/HR2) to form a compact coiled‐coil conformation called 6‐helix bundle or 6HB.^144^, ^147^ Through direct interactions with lipid bilayers, the fusion domain disrupts two apposed membrane layers and fuses the viral envelope to host cell membrane. Ultimately, the viral genome is successfully released into the cytosol of the target cell. In addition to the viral infection through the plasma membrane, the entry of CoVs into cells can be accomplished by the endocytic pathway, depending on the virus strains and host cells.^135^, ^144^

### 3.2 SARS‐CoV

SARS‐Cov, whose intermediary host is the palm civet, is a highly pathogenic respiratory virus that emerged in 2002, leading to global pandemic that affected more than 8000 people in 29 countries.^148^, ^149^ Patients infected with SARS‐CoV initially present “flu‐like” syndrome commonly showing high fever, headache, sore throat, myalgia, and dry cough.^150^, ^151^, ^152^ During the disease progression, ALI or ARDS is developed in a number of patients.^153^ Pathological manifestations can be described as three phases. The first step is the disturbance of gas exchange in the first week, owing to the extensive edema, shedding of ciliated epithelial cells, and deposition of hyaline‐rich substances on the alveolar membrane. In the next step, pulmonary fibrosis occurs that is characterized as the deposition of fibrin at epithelial cells and the alveolar spaces, as well as the infiltration of inflammatory and fibroblasts. Finally, fibrosis of lung tissue, collagen deposition, and proliferation of alveolar and interstitial cells represent the final step of disease, about 6‐8 weeks.^154^, ^155^, ^156^, ^157^, ^158^ Diffuse alveolar damage (DAD) accompanied by hyaline membrane formation as well as interstitial thickening is common characteristics of SARS‐CoV inducing pulmonary damage.^159^ Although many investigators have devoted to inquiring into the pathogenesis of such virus, it has not yet been fully understood until now.

**FIGURE 2:**
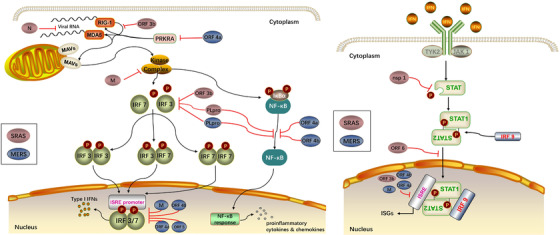
Escape mechanisms of innate immune response of SARS‐CoV and MERS‐CoV

The immune response is the earliest alert system of the host cells warning virus attacks. Ironically, it also aids viral pathogenesis. Pattern recognition receptors (PRRs) that are retinoic acid‐inducible gene I protein (RIG‐I, member of RLRs family) and melanoma differentiation‐associated protein 5 (MDA5) recognize viral pathogen‐associated molecular patterns (PAMPs), such as viral components and replication intermediates, to initiate signaling cascades against virus infection.^160^, ^161^ Once the PAMPs from invaded viruses are detected, RIG‐I and MDA5 interact with the mitochondrial antiviral signaling protein (MAVs) that is a mitochondrial membrane‐bound adaptor molecule, followed by the activation of several kinase complexes and multiple subsequent transcription factors (IRF3, IRF7, and NF‐κB). Activation of NF‐κB induces the production of proinflammatory cytokines and chemokines in the nucleus that is a substantial cause of the ARDS.^162^, ^163^ Phosphorylation of IRF3 and IRF7 by the kinase complexes results in homo‐ or heterodimerization of IRF3 and IRF7. The dimerization initiates the transcription of Type I interferons (IFNs, IFN‐α and IFN‐β), activating the signal transducer and activator of transcription proteins (STATs) to mediate antiviral response (Figure 2).^164^, ^165^, ^166^

Several defensive approaches are used by SARS‐CoV to avoid the PRRs defense system and, ultimately, host innate immune response. One approach is to replicate themselves within the double membrane vesicles (DMVs) that are protected from the PRRs.^167^, ^168^ The eukaryotic mRNAs contain a 5′ cap that usually lacks in the viral mRNAs. However, some viruses such as SARS‐CoV are capable of building the RNA cap through nsp14 and nsp16/nap10 complex, helping them bypass the recognition by PRRs.^169^, ^170^, ^171^, ^172^ In addition, a couple of more proteins encoded by the viruses participate in the suppression of the innate immune response by disrupting the IFN response. Among the nonstructural proteins, nsp 1 mainly involves in the degradation of host mRNAs, inactivation of host translational machinery as well as the inhibition of STAT1 phosphorylating.^173^, ^174^, ^175^ SARS‐CoV PLpro interferes phosphorylation of IRF3 and disrupts NF‐κB signaling probably via interacting with STING.^176^, ^177^, ^178^ The nsp7 and nsp15 are also potential IFN antagonists though the mechanism is not clear.^177^ Structure proteins such as the N protein and M protein are likely to suppress the type I IFN pathways. Because it was known that N protein inhibits the IFN transcription, the N protein could have a strong potential influence on the viral RNA.^179^ M protein blocks the formation of signaling kinase complexes, and suppresses the IRF3 and IRF7 activities, suggesting the potential role of the N protein in the viral infection as well.^180^ Such accessory proteins as ORF 3b protein are able to inhibit the RIG‐I activity and IRF3 phosphorylation in addition to the transcription of IFN‐stimulated genes (ISGs) via the ISRE promoter, while ORF6 blocks the nuclear translocation of STAT1.^181^, ^182^ In spite of the number of research findings in vitro, they have not been validated in vivo. Therefore, it is urgent to examine the findings in vivo for a clear and solid understanding of the infectious process.

Besides the immune response of the host, ACE2 also plays an essential role in the pathogenesis of SARS‐CoV. As a negative regulator on the RAS, ACE2 has been closely linked to the pathogenesis of pulmonary diseases and considered as a protective factor for ALI.^113^ Consequently, the downregulation of ACE2 mediated by SARS‐CoV binding might give an explanation for the progression of severe lung damage occurred on some SARS patients.^114^

### 3.3 MERS‐CoV

A decade after SARS, another novel CoV was identified as the pathogen of MERS that caused a higher mortality rate (30%‐40%) compared with SARS (around 10%).^183^, ^184^ SARS‐CoV and MERS‐CoV are both emerged from bats, and are disseminated to human through palm civets and dromedary camels, respectively.^185^, ^186^, ^187^, ^188^ MERS‐CoV and SARS‐CoV share some common clinical manifestations ranging from asymptomatic to severe pneumonia in multiple organs,^150,184,189^ and pathological features including inflammatory cells infiltration and DAD.^183^ Similar to SARS‐CoV, MERS‐CoV is also capable of causing immune dysregulation by attenuating the innate immune response (Figure 2).^190^, ^191^

Type I interferon is important for the inhibition of MERS‐CoV replication in host cells probably via the suppression of the DMVs formation.^192^, ^193^ Capping viral mRNA by nsp14 and nsp16/nap10 complex protects MERS‐CoV, as well as SARS‐CoV, from the PRRs recognition since the structure of nsp16/nap10 complex in both viruses are analogous.^194^ Besides, several proteins of MERS‐CoV are involved in immune escape mechanism by involving in the signaling cascades. It was reported that IRF3 nuclear translocation and IFN promoter activation are inhibited by M protein, ORF4a, ORF4b, and ORF5, former three of which also restrained the expression from an the ISRE promoter.^195^ Inhibition of the phosphorylation of IRF3 might be the mechanism for IFR3 translocation inhibiting.^194^ Moreover, MERS‐CoV ORF4a can interact with IFN‐inducible double‐stranded DNA (dsDNA) –dependent protein kinase activator A (PRKRA) and subsequently control the function of RIG‐I and MDA5, resulting in the disruption of the IFN response.^196^, ^197^ ORF4a and ORF4b are thought to participate in the NF‐κB signaling by downregulating the gene stimulated by NF‐κB and affecting the kinase complexes, respectively.^198^ Functions of PLpro and nsp1 of MERS‐CoV are analogous to the functions of those in SARS‐CoV.^199^, ^200^

Like ACE2, the entry receptor DPP4 for MERS‐CoV has also a pivotal role in the disease pathogenesis and is considered as a key factor for the intraspecies variation shown in MERS infection.^201^, ^202^ It is usually expressed in type II pneumocytes that cover 2% of the alveolar surface.^115^, ^124^ Approximately 95% of the surface area is occupied by the type I pneumocytes that are responsible for gas exchange.^203^, ^204^ But the autopsy reports indicated that both type I and II pneumocytes in patients died from MERS‐harbored DPP4 expression and these pneumocytes were infected by the virus.^205^, ^206^ MERS‐CoV infection of type I pneumocytes might lead to the damage of the cells in the alveoli subsequently causing the DAD.^207^ It suggests that type I pneumocytes expressing DPP4 might be included in the pathogenesis of the disease. This might explain why chronic obstructive pulmonary disease (COPD) patients attacked by MERS‐CoV had poor outcomes, since the expression of DPP4 was predominantly upregulated on type I pneumocytes in such patients.^202^, ^208^ Besides, owing to high expression of DPP4 shown in the kidney, the renal dysfunction might be caused by either the direct infection or the hypoxic damage.^116^ Evidence of tubular injury, such as cell debris and tubular dilation, could be observed in the late stage of the infection in MERS‐CoV‐infected mice. However, no virus could be detected in such animals in the early stage after infection, meaning such pathologic changes might be related to the hypoxia.^125^

### 3.4 SARS‐COV‐2

The outbreak of COVID‐19, whose pathogen is SARS‐CoV‐2, now poses a serious threat to the global public health.^209^, ^210^ Since the emergence of the virus, SARS‐CoV‐2 has affected more than 14 million people with more than 597 thousand deaths worldwide as of July 2020. Next‐generation sequencing of the novel virus has been developed soon after the outbreak, indicating that SARS‐CoV‐2 is closely related to the bat‐derived SARS‐like CoVs.^107^, ^211^ It is now believed that bats are likely to be the natural reservoir,^212^, ^213^ and pangolins are regarded as intermediary host according to the later studies. Typical clinical presentations of SARS‐CoV‐2‐infected patients include fever, dyspnea, dry cough fatigue, myalgia, pneumonia, and ARDS symptoms, similar to those of SARS and MERS patients.^214^, ^215^, ^216^ However, intestinal disorders such as diarrhea are less frequent in COVID‐19 patients than SARS.^214^, ^215^, ^217^ Furthermore, in spite of the variation of amino acid at some residues, homology modeling informed that SARS‐CoV‐2 and SARS‐CoV have an analogous RBD structure, and share the same target cell receptor, ACE2, to mediate the viral entry.^107^, ^108^, ^216^, ^218^, ^219^, ^220^ It is speculated that ACE2 is involved in the pathogenesis of SARS‐CoV‐2. Owing to the current sparsity of data on the pathological characters of SARS‐CoV‐2, it is poorly understood. A case report from an infected patient died of this disease showed that DAD with hyaline membrane formation, infiltration of inflammatory cells, and pulmonary edema could be found in the samples taken from their lungs, which is notably corresponding with the symptoms of SARS and MERS patients.^221^ Additionally, lymphopenia is a common manifestation in COVID‐19 patients and might be an effective indicator to estimate the severity of hospitalized COVID‐19 patients.^222^ Lymphopenia is also supposed to be a vital factor related to the pathogenesis that has not been elucidated so far.^213^ Moreover, the concentration of some cytokines and chemokines detected in the plasma was higher in COVID‐19 patients compared with healthy individuals. Moreover, higher plasma levels of GSCF, IL‐2, IL‐7, IL‐10, MCP1, MIP1A, IP10, and TNF‐α were linked to the more severe disease.^215^ All of the data reveal that immunopathology may occupy a crucial place in the development of the disease, and further researches about the pathogenesis of SARS‐CoV‐2 are urgently needed in the future.

## 4 TREATMENTS AND INTERVENTIONS AGAINST CORONAVIRUS

### 4.1 Current antivirus therapeutic mechanisms

Owing to the fact that no effective specific antiviral therapies are currently available for SARS, MERS, and COVID‐19, isolation and symptomatic support cares are the major management strategies for suspected and confirmed cases requiring hospital treatment including oxygen inhalation, fluid management, and rational use of antibiotics, to prevent organ failure and secondary bacterial infection and alleviate the symptoms.^189^, ^223^, ^224^, ^225^ Thus, the identification of effective agents against human CoVs is urgently needed in the response to the current COVID‐19 outbreak.

**FIGURE 3:**
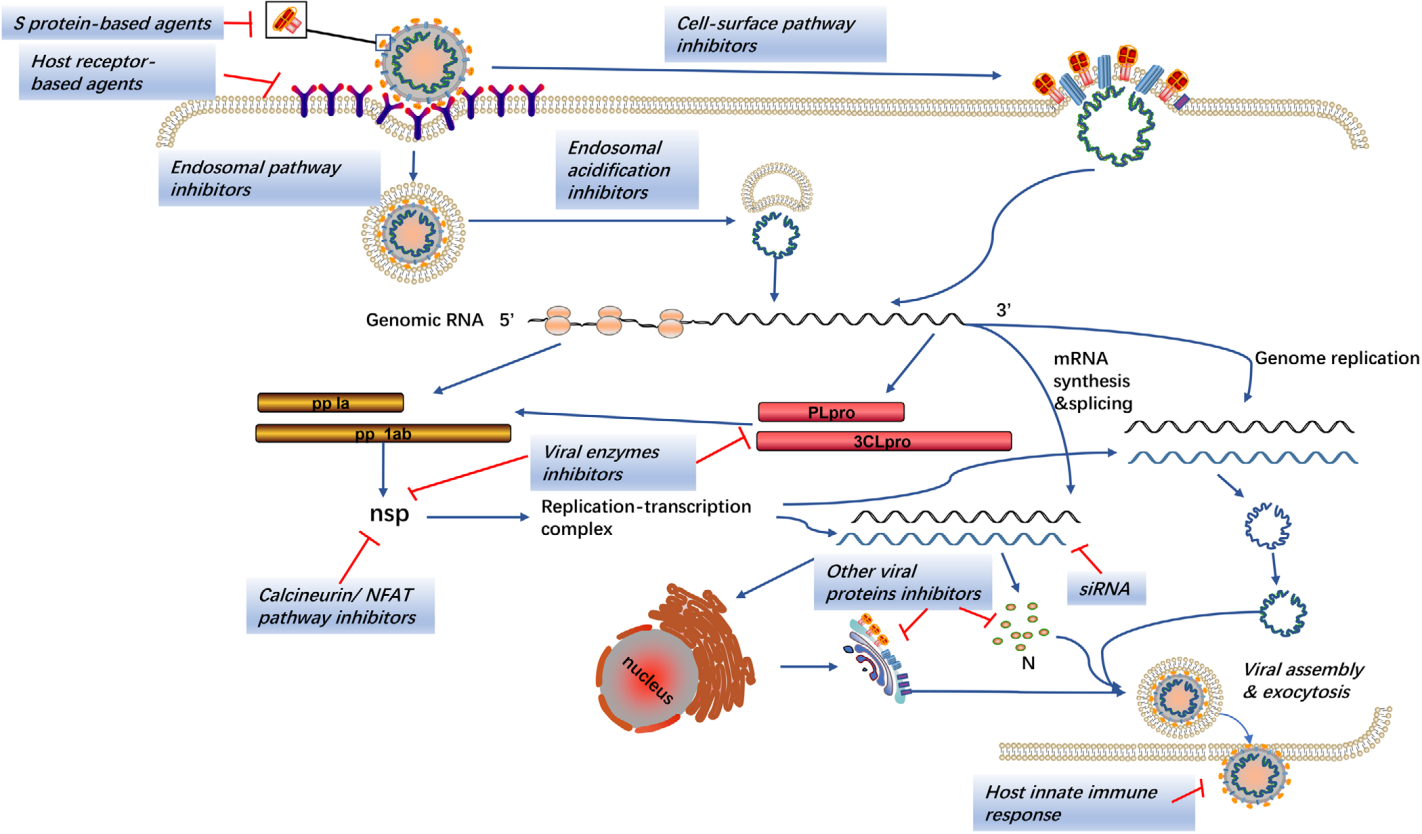
Mechanisms of current anti‐CoV therapeutic agents

All of SARS‐CoV, MERS‐CoV, and SARS‐CoV‐2 encode structure proteins (like S protein), nonstructure proteins (eg, PLpro, 3CLpro, RdRp, and helicase), and accessory proteins that are essential for the viral life cycle and that are considered as important targets for the development of antiviral agents (Figure 3).^226^, ^227^ Analyses of genomic sequences and protein structure indicated that the catalytic sites of four crucial enzymes and the key drug‐binding pockets in viral enzymes were conserved across SARS‐CoV, MERS‐CoV, and SARS‐CoV‐2.^227^ Therefore, the therapeutic experience based on SARS and MERS is capable to guide the treatment of COVID‐19.

The idea to disturb the normal life cycle of the virus provides significant insights into the clinical treatment strategy. The S protein is important for the discovery of antiviral agents due to its multifunction in virus infection. RBD located on the S1 subunit can bind to the host cell receptors (ACE2 for SARS‐CoV and SARS‐CoV‐2, DPP4 for MERS‐CoV) initiating the conformational changes in S2 subunit to get viral and cell membranes closer and trigger membrane fusion.^228^ Consequently, the interaction between RBD and the host cell receptors serves as a key target for the production of neutralizing antibody followed by the vaccine development.^31^, ^229^ Monoclonal antibodies (mAbs) and fusion inhibitors against S1 and S2 subunit, respectively, are potential antivirus drugs to conquer the viral infection, and the agents targeting the host receptors also play a similar role.^230^, ^231^, ^232^, ^233^, ^234^ Likewise, cleavage at the protease site of the S1/S2 complex by host proteases such as TMPRSS2 and furin is necessary for the membrane fusion and syncytium formation.^143^, ^235^ The endosomal cysteine protease cathepsins promote the entry of CoVs into the host cell via the endosomal pathway.^236^ Inhibitors of these host proteases can potently block the cell entry, which has been proved in vitro and require further validation on animals studies.^237^

Once entering the host cells, CoVs release the nucleocapsid and genomic RNA into the cytoplasm and start the translation of the replicase gene. The large replicase pp1a and pp1ab are cleaved by PLpro and 3CLpro to produce nonstructural proteins like RdRp and helicase, forming the replication‐transcription complex.^238^ Numerous agents inhibiting these proteins have shown anti‐CoV effects in vitro. Combination of the hydrophobic domains of the replication‐transcription complex to the endoplasmic reticulum membrane can form the typical CoV replication structures such as DMVs and convoluted membranes, protecting CoVs from the detection of PRRs.^168^, ^239^ Viral RNA synthesis produces genomic and subgenomic RNAs. Then the subgenomic RNAs are translated to generate the structural and accessory proteins, participating in the assembly of the virion that is released into the extracellular compartment via exocytosis.^8^ Small interfering RNAs (siRNAs) disturbing these processes could be used in the treatment of CoVs infections.

Although CoVs are capable of disturbing the IFN response, they are still sensitive to the IFN treatment in vitro, indicating that augmented host innate IFN response can be an effective strategy to control the viral infection.^207^, ^240^, ^241^, ^242^ In addition to the enhancement of INF response, several other cell signaling pathways are also regarded as potential anti‐CoV targets. These include calcineurin‐nuclear factor of activated T cells (NFAT) pathway and kinase signaling pathways such as ERK/MAPK and PI3K/AKT/mTOR pathways, the inhibitors of which have exhibited anti‐CoV activities as well.^243^, ^244^, ^245^

Since the discovery of new interventions may take months or even years, a more efficient approach is to repurpose existing antiviral agents approved for treating related viral infections. The followings are approved drugs or preclinical compounds that have potential antiviral abilities against SARS, MERS, and COVID‐19.

### 4.2 Virus‐targeted therapeutic strategies

#### 4.2.1 Agents based on viral enzymes

All of the major proteases of the virus are attractive druggable targets since they are essential for viral transcription and replication (Table 2). As a key part of replication‐transcription complex, RdRp participates in the production of genomic RNA and subgenomic RNA. Nucleoside analogues targeting RdRp is capable of inhibiting viral RNA synthesis in a great variety of RNA viruses including CoVs.^246^, ^247^, ^248^, ^249^, ^250^ Favipiravir (T‐705), a guanine analogue approved in Japan for influenza treatment, has been proven to effectively interfere the RNA synthesis of RNA viruses such as influenza virus, Ebola virus, and other hemorrhagic fever viruses at RdRp level.^251^, ^252^, ^253^, ^254^, ^255^, ^256^ Several studies concluded that favipiravir could inhibit avian influenza A (H5N1) virus and Ebola virus infection in mice.^256^, ^257^ Also, favipiravir has been proved with a notable effect increasing the survival rate of Ebola‐infected patients from 35.3% to 56.4% in Sierra Leone.^258^ A recent study ended with the statement that favipiravir owns the ability against SARS‐CoV‐2 (EC50 = 61.88uM, CC50 > 400uM, SI > 6.46).^259^ COVID‐19 patients were enrolled in randomized trials for the evaluation of the efficacy of favipiravir plus INF‐α or baloxavir marboxil (ChiCTR2000029544 and ChiCTR2000029600). Another guanosine derivative with broad‐spectrum antiviral activity, ribavirin, has been authorized for HCV and respiratory syncytial virus (RSV) treatment.^260^ Accurate mechanism of ribavirin against virus infection is not clear, but inhibition of mRNA capping and viral RNA synthesis could be pivotal to its antiviral activity.^261^ Although high dose of ribavirin has been applied to SARS treatment, the anti‐MERS‐CoV effects were moderate at such dose in rhesus macaques infected by MERS‐CoV and no obvious survival benefit has been observed in MERS patients.^225^, ^262^, ^263^, ^264^, ^265^, ^266^, ^267^ Recently, an open‐label, randomized phase II clinical trial (NCT04276688) has revealed that triple combination of ribavirin, interferon, and lopinavir‐ritonavir in COVID‐19 treatment was safe and superior to lopinavir‐ritonavir therapy alone in remission of symptoms, shortening virus shedding and promoting discharge of mild to moderate COVID‐19 patients.^268^ Remdesivir (GS‐5734) is a small‐molecule monophosphoramidate prodrug of an adenosine analog with the ability to interfere with the RNA polymerase and the proofreading exoribonuclease and terminate the nonobligate chain.^269^, ^270^, ^271^ Currently developed for the treatment of Ebola virus infection, remdesivir shows a potential antivirus activity to a diverse panel of RNA viruses including SARS‐CoV, MERS‐CoV, RSV, Nipah virus (NiV), and Hendra virus in vitro and/or in vivo.^270^, ^272^, ^273^ Besides, it demonstrated broad‐spectrum anti‐CoV capacity showing the effective suppression to the epidemic and endemic CoVs, and might be effective against the emerging CoVs now and in the future.^272^ As to the ongoing COVID‐19, clinical and preclinical researches have been set up to investigate the efficacy of remdesivir to the emerging CoV, which indicated that remdesivir was able to inhibit SARS‐CoV‐2 in cultured cells (EC50 = 0.77 µM in Vero E6 cells) and a US COVID‐19 patient recovered after treating with remdesivir intravenously.^209^, ^259^ Moreover, two phase III clinical trials (NCT04252664 and NCT04257656) planning to enroll 308 and 453 participants, respectively, have been initiated to confirm the value of intravenous remdesivir in COVID‐19 patients with the intervention of 200 mg on day 1 and 100 mg once‐daily maintenance for 9 days. However, the first clinical trial (NCT04252664) has been suspended so far and the second trial (NCT04257656) with 237 COVID‐19 patients enrolled finally indicated that remdesivir hardly shown any statistically significant clinical benefits.^274^ Conversely, a research found that 36 of 53 (68%) hospitalized patients suffered from severe COVID‐19 and treated with compassionate‐use remdesivir could achieve clinical improvement.^275^ In addition, a phase III, randomized, double‐blind, placebo‐controlled trial (NCT04280705) conducted by Beigel et al uncovered the fact that remdesivir prevailed over placebo in shortening the time to recovery in adults patients.^276^ Though remdesivir has been approved by the Food and Drug Administration (FDA) to treat severe COVID‐19 patients, further researches are urgently required to determine the efficacy and the indication of remdesivir therapy. A novel synthesized nucleoside analogue, BCX4430 (Galidesivir), is designed to inhibit viral RNA polymerase activity via terminating nonobligate RNA chain.^277^ BCX4430 exhibits a promising antiviral capability against a wide array of RNA viruses including filoviruses (Ebola virus and Marburg virus) and CoVs (SARS‐CoV and MERS‐CoV).^277^ It is currently tested in phase I clinical trial (NCT03800173) for Marburg virus and can be a potential countermeasure against viral diseases that threaten the public health in the world. A recent study also concluded that penciclovir, another RdRp inhibitor that is approved for HSV, showed effects on reducing SARS‐CoV‐2 infection by high‐dose administration (EC50 = 95.96 µM, CC50 > 400 µM, SI > 4.17).^259^ Although resistance to nucleoside analogs has rarely been reported, it is worth noting that mutation in RdRp is probably responsible for the acquired resistance and should be monitored for the possible resistance.^278^

**TABLE 2:** Virus‐targeted agents for HCoVs

Targets	Agents	Mechanisms	Antiviral spectrum	Status	Refs.
**RdRp**	Favipiravir (T‐705)	Interfering with RNA synthesis of RNA virus at RdRp level	SARS‐CoV‐2	Randomized trials for COVID‐19 in combination with INF‐α or baloxavir marboxil (ChiCTR2000029544 and ChiCTR2000029600)	^249^, ^250^, ^251^, ^252^, ^253^, ^254^, ^255^, ^256^, ^257^
	Ribavirin	Inhibiting mRNA capping and viral RNA synthesis	SARS‐CoV MERS‐CoV SARS‐CoV‐2	Randomized trial for SARS (NCT00578825) Randomized for COVID‐19 in combination with a PEGylated interferon (ChiCTR2000029387) Phase II randomized trial for COVID‐19 in combination with interferon beta‐1b and lopinavir‐ritonavir (NCT04276688)	^224^, ^258^, ^259^, ^260^, ^261^, ^262^, ^263^, ^264^, ^265^, ^268^
	Remdesivir (GS‐5734)	Interfering the RNA polymerase and the proofreading exoribonuclease and terminating the nonobligate chain	SARS‐CoV MERS‐CoV SARS‐CoV‐2	Phase III clinical trials for COVID‐19 (NCT04252664, NCT04257656, NCT04280705)	^208^, ^257^, ^266^, ^267^, ^268^, ^269^, ^270^
	Galidesivir (BCX4430)	Inhibiting viral RNA polymerase activity via terminating nonobligate RNA chain	SARS‐CoV MERS‐CoV	Phase I clinical trial for Marburg virus (NCT03800173)	^257^, ^271^
	Penciclovir	RdRp inhibitor	SARS‐CoV‐2	Applied in HSV	^257^
	Acyclovir fleximer analogues	RdRp inhibitor	HCoV‐NL63 MERS‐CoV	Preclinical	^248^
**PLpro**	Disulfiram	PLpro inhibitor	SARS‐CoV MERS‐CoV	Applied in chronic alcohol dependence	^281^
	6‐mercaptopurine and 6‐thioguanine	Thiopurine analogues that inhibits PLpro	SARS‐CoV MERS‐CoV	Preclinical	^277^
	Compound 6	Inhibiting MERS PLpro	MERS‐CoV	Preclinical	^278^
**3CLpro**	Lopinavir / ritonavir	3CLpro inhibitors	SARS‐CoV MERS‐CoV SARS‐CoV‐2	Applied in HIV Phase II/ III clinical trial for MERS (NCT02845843) Phase III clinical trials for COVID‐19 (NCT04251871, NCT04255017,NCT04252274, ChiCTR2000029539, ChiCTR2000029308, NCT04261270)	^282^, ^283^, ^284^, ^285^
	Darunavir and cobicistat	3CLpro inhibitors	SARS‐CoV‐2	Applied in HIV Phase III clinical trial for COVID‐19 (NCT04252274)	^‐^
	ASC09F	3CLpro inhibitors	SARS‐CoV‐2	• Phase III clinical trial for COVID‐19 (NCT04261270)	^‐^
	GC376, GC813, SK80, Peptidomimetic Inhibitors (Compound 6), Neuraminidase inhibitor analogues (compound 3k),	3CLpro inhibitors	SARS‐CoV MERS‐CoV	Preclinical	^377^, ^378^, ^379^, ^380^, ^381^
**Helicase**	Bananins and 5‐ Hydroxychromone derivatives	Inhibiting both the ATPase and helicase activities	SARS‐CoV	Preclinical	^286^
	Triazole derivatives (Compound 16)	Inhibiting both the ATPase and helicase activities	MERS‐CoV	Preclinical	^287^
	SSYA10‐001 and ADKs	Inhibiting helicase without affecting ATPase activity	SARS‐CoV MERS‐CoV	Preclinical	^288^
**S protein**	Nafamostat	Inhibiting s protein‐mediated membrane fusion	MERS‐CoV SARS‐CoV‐2	• Applied in anticoagulant therapy in Asian countries	^257^
	Griffithsin	Specially binding to oligosaccharides located on the surface of viral glycoproteins, leading to inhibition of viral entry	HCoV‑229E HCoV‑OC43 SARS‐CoV, MERS‐CoV	Preclinical	^289^, ^290^
	HR2P, HR2P‐M2, HR1P, HR1M, HR1L, HR2L, MERS‐5HB	Targeting the S2 subunite of S protein of MERS‐CoV, thus inhibiting the S protein‐mediated membrane fusion	MERS‐CoV	Preclinical	^227^, ^293^, ^294^, ^382^, ^383^
	Peptides (P9)	Inhibiting S protein‐mediated membrane fusion	SARS‐CoV MERS‐CoV	Preclinical	^384^
	siRNA	Short chains of dsRNA interfering the expression of S protein	Narrow‐spectrum	Preclinical	^295^, ^296^, ^297^
**M, N, E, and accessory proteins**	siRNA	Inhibiting the replication of CoVs via silencing M, N, E, ORF3a and ORF7a/7b	Narrow‐spectrum	Preclinical	^298^, ^299^
	Resveratrol	Deregulating the expression of N protein and the apoptosis induced by MERS‐CoVs	MERS‐CoV	Preclinical	^300^, ^301^, ^302^, ^303^, ^304^, ^305^, ^306^
	Hexamethylene amiloride	Suppressing the ion channel activity of E protein of CoVs	SARS‐CoV HCoV‐229E	Preclinical	^307^
**DNA** **metabolism**	Gemcitabine hydrochloride	Inhibitor of DNA metabolism	SARS‐CoV MERS‐CoV	Preclinical	^308^
**Lipid membrane**	LJ001 and JL103	Inhibiting membrane fusion via mediating the changes of membrane properties, including the decrease in membrane fluidity	Broad‐spectrum against enveloped viruses	Preclinical	^309^
**Host cell membrane‑bound viral replication complex**	K22	Inhibiting membrane‑bound RNA synthesis and double membrane vesicle formation	HCoV‐229E SARS‐CoV MERS‐CoV	Preclinical	^385^
**Others**	Arbidol	Unclear	SARS‐CoV‐2	Phase IV clinical trials for COVID‐19 (NCT04260594, NCT04254874, NCT04255017)	–
	Oseltamivir	Unclear	SARS‐CoV‐2	Phase IV clinical trial for COVID‐19 (NCT04255017) Phase III clinical trial for COVID‐19 (NCT04261270)	–

Abbreviations: CoV, coronavirus; MERS, Middle East respiratory syndrome; PLpro, papain‐like cysteine protease; RdRp, RNA‐dependent RNA polymerase; SARS, severe acute respiratory syndrome; 3CLpro, 3C‐like serine protease.

CoVs PLpro enzymes display proteolytic, deubiquitylating, and deISGylating activities.^279^, ^280^, ^281^ PLpro was first regarded as a druggable target for SARS‐CoV, and then several compounds targeting SARS‐CoV PLpro were also found to be effective against MERS‐CoV PLpro, recently.^282^, ^283^ Though numerous PLpro inhibitors have been identified, many of them only inhibit enzymatic activities and do not affect on the viral replication.^284^, ^285^ A study from Lin et al suggested that an FDA‐approved alcohol‐aversive drug, disulfiram could inhibit SARS‐CoV and MERS‐CoV PLpro via different mechanisms. And the synergistic inhibition between disulfiram and known PLpro inhibitors, like 6‐thioguanine and mycophenolic acid, to MERS‐CoV might offer the potential combination treatments against CoVs in clinical.^286^

Another essential protease that cleaves the viral polyproteins during viral replication is 3CLpro. Similar to PLpro, a great many of inhibitors have been identified with the ability to target CoVs 3CLpro. Among the 3CLpro inhibitors, the human immunodeficiency virus (HIV) protease inhibitors are the most comprehensively studied such as lopinavir, ritonavir, ASC09F, as well as darunavir and cobicistat. Lopinavir and ritonavir, applied in combination to treat HIV infection, have shown improvement in the outcome of SARS patients in nonrandomized trials.^287^, ^288^ Though lopinavir alone hardly displayed antiviral activity against MERS‐CoV in vitro, the combination of lopinavir and ritonavir ameliorated the outcome in MERS‐CoV‐infected nonhuman primates.^289^, ^290^ Therefore, the efficacy of the lopinavir‐ritonavir combination in MERS patients should be reappraised (NCT02845843). However, no benefit was observed in lopinavir‐ritonavir treatment compared to standard care in a randomized, controlled, open‐label clinical trial (ChiCTR2000029308) involving severe COVID‐19 patients.^291^ Further trials are still needed to confirm the therapeutic efficacy. In addition, several other clinical trials have been developed to confirm the efficacy of 3CLpro inhibitors on COVID‐19 (NCT04252274, NCT04251871, NCT04255017, ChiCTR2000029539, NCT04251871, NCT04255017, and NCT04261270), as well as darunavir and cobicistat (NCT04252274), ASC09F combined with oseltamivir (NCT04261270).

Helicase acts on the duplex oligonucleotides and turns them into single strands in an ATP‐dependent manner in the CoV replication cycle. That helicases in different CoVs are highly homologous making them potentially strong targets for the CoVs therapeutic options. Based on the mechanism actions, the helicase inhibitors can be approximately classified into two groups. One is able to inhibit both the ATPase and helicase activities represented by bananins derivatives, 5‐hydroxychromone derivatives, and triazole derivatives (compound 16).^292^, ^293^ The other group including SSYA 10‐001 and ADKs has the ability to inhibit the helicase activity with no or little effects on the ATPase activity.^294^ However, the toxicity of helicase inhibitors needs to be examined before being applied to human patients.

#### 4.2.2 Agents based on viral structure proteins

The transmembrane glycoprotein, S protein, is also a promising target for antiviral agents’ development (Table 2). One class of antiviral drugs targeting S protein mostly blocks the spike‐mediated membrane fusion. A potent MERS‐CoV inhibitor, nafamostat, has demonstrated to be inhibitive against the SARS‐CoV‐2 infection (EC50 = 22.50 µM, CC50 > 100 µM, SI > 4.44).^259^ Griffithsin is a red‐alga‐derived lectin, which specially binds to oligosaccharides located on the surface of viral glycoproteins, for example, S glycoprotein of SARS‐CoV and HIV glycoprotein 120.^295^ A wide range of human CoVs infection could be inhibited by griffithsin, comprising HCoV‐229E, HCoV‐NL63, HCoV‐OC43, and SARS‐CoV.^295^, ^296^ But the value of griffithsin in the treatment or prevention of COVID‐19 is needed to be evaluated urgently. S2 subunit of S protein contains two heptad repeat (HR1 and HR2). Antiviral peptides analogous derived from these regions exhibited inhibition to the spike protein‐mediated cell‐cell fusion and viral entry in viruses such as SARS‐CoV, MERS‐CoV, as well as HCoV‐229E.^231^, ^297^, ^298^ HR2P, a peptide spanning HR2 sequences of MERS‐CoV S protein, was capable of interacting with HR1 region to form a 6‐HB complex, resulting in potent inhibition of viral fusion and replication. Its analog HR2P‐M2 exhibited an obvious improvement in stability, solubility, and antiviral activity after being modified with hydrophilic residues.^228^ Additionally, HR2P‐M2 intranasal administration effectively prevented experimental mice transduced by adenoviral vectors conveying human DPP4 from MERS‐CoV infection with a >1000‐fold decrease in viral titers in the lung, and this protection was intensified via the combination of HR2P‐M2 and IFN‐β.^299^ Another newly designed fusion inhibitor from MERS‐CoV called MERS‐five‐helix bundle (MERS‐5HB), which is derived from the 6‐HB, also displayed a potent suppression on S protein‐mediated syncytial formation. Compared with MERS‐6HB, MERS‐5HB lacks one HR2, which endows its capability to interact with viral HR2 to interrupt the membrane fusion.^300^ Besides, MERS‐5HB could effectively inhibit the entry of pseudotyped MERS‐CoV with 50% inhibitory concentration (IC50) about 1 µM.^300^ Altogether, the resistance of these drugs can be overcome by combining antiviral peptides aiming at various domains of S2 subunit, which may also attain synergistic effects in vitro. As for siRNAs, which displayed antiviral activities in vitro as well as in SARS‐CoV‐infected rhesus macaques, are still under preclinical development and demand further studies to seek out the reliable drug delivery methods in a human before the clinical application.^301^, ^302^, ^303^

M, N, and E proteins and some accessory proteins are not only vital to the virion assembly but also involved in viral pathogenesis in which they function in the interruption of host innate immune response to assist viral infection. For instance, M protein as well as accessory proteins 4a, 4b, and 5 of MERS‐CoV act as IFN antagonist, and N protein of SARS‐CoV serves as an inhibitor of viral RNA. Researches carried out by He et al and Akerstrom et al emphasized that siRNAs silencing M, N, E, ORF3a, and ORF7a/7b play an important role in the inhibition of viral replication of the SARS‐CoV.^304^, ^305^ But the delivery methods still limit their application in human being. Nevertheless, various agents related to these proteins are discovered via functional analysis. One example is resveratrol, a natural stilbene derivative demonstrated to reduce inflammation and exert antiviral activity against multiple viruses.^306^, ^307^, ^308^, ^309^, ^310^, ^311^ In addition, it exhibited significant inhibition of MERS‐CoV infection and prolonged cellular survival after virus challenge in vitro via deregulating the expression of N protein and the apoptosis induced by MERS‐CoV.^312^ Alternatively, hexamethylene amiloride, a viroporin inhibitor, is capable to suppress the ion channel activity of E protein of CoVs such as HCoV‐229E and SARS‐CoV.^313^ Identified as DNA metabolism inhibitor, gemcitabine hydrochloride is a deoxycytidine analog inhibiting both SARS‐CoV and MERS‐CoV with micromolar EC50 and low toxicity, which suggests its potential antiviral capacity for other human CoVs.^314^ LJ001 and JL103, two novel lipophilic thiazolidine derivatives, could induce several changes in membrane properties including the decrease in membrane fluidity, contributing to inhibition of membrane fusion, which made them become broad‐spectrum enveloped virus entry inhibitors and potential anti‐CoV agents.^315^

### 4.3 Host‐cell‐targeted therapies

#### 4.3.1 Enhancement of host innate immune response

The host innate immune response is vital to the interruption of viral replication. Recombinant interferons have been applicated in treating various viruses as well as many CoVs (Table 3). Though the host interferon response can be inhibited by the CoVs, they are still proved effective against CoVs infection such as SARS‐CoV and MERS‐CoV in vitro and several animal models.^242^, ^264^, ^289^, ^299^, ^316^ Recombinant interferons are usually combined with other antiviral agents including ribavirin or lopinavir/ritonavir to treat SARS‐CoV and MERS‐CoV infections,^290^, ^317^ and the anti‐CoVs efficacy of interferons is enhanced when added with ribavirin.^318^ A combination of interferon‐α2b with ribavirin reduced virus replication and improves clinical outcomes in a rhesus macaque model of MERS.^264^ However, the effectiveness of combination treatments comprising interferons and ribavirin is still controversial in clinical researches. A study of five patients received interferon‐α2b and ribavirin showed no survival, but the finding might be not reliable owing to the delayed administration.^266^ Contrarily, in another study (*n* = 44), mortality rates of individuals receiving interferon‐α2b and ribavirin exhibited a significant reduction in day 14, compared with the individuals received standard supportive care, but no significant difference was observed in day 28.^265^ Moreover, no significant difference in mortality rates between interferon‐α2b and ribavirin treatment group and interferon‐β1a and ribavirin treating group was observed in a retrospective study.^267^ On the other hand, interferon‐β1b displayed stronger inhibition to the MERS‐CoV replication in vitro compared with other interferons, and combined use of interferon‐β1b with other antiviral compounds should be evaluated in further research.^289^, ^290^

**TABLE 3:** Host‐targeted agents for HCoVs

Targets	Agents	Mechanisms	Antiviral spectrum	Status	Refs.
**Host interferon response**	Recombinant interferons	Exogenous interferons augmented host innate IFN response against CoVs infection	SARS‐CoV MERS‐CoV SARS‐CoV‐2	Marked Randomized trial for COVID‐19 (NCT04251871, ChiCTR2000029638)	^284^, ^285^, ^311^, ^312^
	Nitazoxanide	Inducing the host innate immune response via intensifying interferon‐α and interferon‐β production through fibroblasts and activation of protein kinase R (PKR)	SARS‐CoV‐2	Marked	^313^, ^314^, ^315^
	Polyinosinic:polycytidylic acid (poly(I:C))	Type I interferon inducer	MERS‐CoV	Phase II clinical trial for malignant gliomas patients Preclinical research for MERS‐CoV	^191^, ^316^, ^317^
**Host receptor**	N‐(2‐aminoethyl)‐1‐aziridine‐ethanamine (NAAE), P4 and P5	ACE2 inhibitors blocking cell‐cell membrane fusion and viral entry	SARS‐CoV SARS‐CoV‐2	Marked	^318^, ^319^
	YS110 and Anti‑DPP4 mAb clones 2F9	DPP4 inhibitors blocking cell‐cell membrane fusion and viral entry	MERS‐CoV	Phase I clinical trial of YS110 for patients with advance solid tumors Preclinical research of mAbs clones 2F9 for MERS‐CoV	^320^
**Host proteases**	K11777	Cathepsin inhibitors inhibiting the endosomal pathway, thus blocking viral entry	SARS‐CoV MERS‐CoV	Preclinical	^236^, ^321^, ^322^
	Camostat mesylate	TMPRSS2 inhibitor interrupting the TMPRSS2‐mediated cell surface entry	HCoV‐229E SARS‐CoV MERS‐CoV	Preclinical	^323^, ^324^
	dec‑RVKR‑CMK	Furin inhibitor inhibiting furin‑mediated cleavage of S	MERS‐CoV	Preclinical	^143^
**Endocytosis**	Chlorpromazine, triflupromazine, fluphenazine	Inhibiting clathrin‐mediated endocytosis via affecting the assembly of clathrin‐coated pits at the plasma membrane	SARS‐CoV MERS‐CoV	Approved as antipsychotic agents	^308^, ^326^
	Ouabain and bufalin	Binding sodium/potassium‐transporting ATPase subunit α1 (ATP1A1)	MERS‐CoV	Approved as cardiotonic steroids	^326^
	Chloroquine, Hydroxychloroquine	Increasing endosomal pH, thus affecting viral infection	HCoV‐229E HCoV‐OC43 SARS‐CoV MERS‐CoV SARS‐CoV‐2	Approved as autoimmune disease and antimalarial agents Open‐label trial for COVID‐19 (ChiCTR2000029609) Phase II clinical trial of chloroquine for COVID‐19 (NCT04323527) Phase III clinical trial of hydroxychloroquine for COVID‐19 (NCT04334148)	^257^, ^327^, ^328^, ^329^, ^330^, ^331^, ^332^, ^333^
**Other antiviral agents**	Cyclosporin A (CsA), alisporivir	Cyclophilin inhibitors affecting the calcineurin/NFAT pathway, blocking the viral replication	HCoV‑NL63 HCoV‑229E SARS‐CoV MERS‑CoV	Marked	^243^, ^244^, ^335^, ^336^, ^337^
	Selumetinib, Trametinib, Rapamycin	Inhibiting the ERK/MAPK and PI3K/AKT/mTOR signaling pathways	SARS‐CoV MERS‐CoV	Marked	^242^
	Dexamethasone, Methylprednisolone	‐	SARS‐CoV MERS‐CoV SARS‐CoV‐2	Marked Clinical trials for COVID‐19 patients (ChiCTR2000029386, ChiCTR2000029656, ChiCTR2000029656, NCT0424459)	^350^, ^351^, ^352^, ^353^, ^354^, ^355^

Abbreviations: ACE2, Angiotensin‐converting enzyme 2; CoV, coronavirus; DPP4, dipeptidyl peptidase IV; SARS, severe acute respiratory syndrome; MERS, Middle East respiratory syndrome.

Nitazoxanide, originally identified as an antiprotozoal agent, was later considered as a broad‐spectrum antiviral agent able to inhibit the replication of numerous DNA and RNA viruses such as RSV, parainfluenza, rotavirus, HBV, HCV, HIV, yellow fever, as well as CoVs in vitro.^319^ Several clinical trials have confirmed its potential value in treating influenza, chronic HBV, and HCV.^319^, ^320^, ^321^ Moreover, recent research indicated that nitazoxanide was capable of inhibiting SARS‐CoV‐2 infection at a low micromolar concentration (EC50 = 2.12 µM; CC50 > 35.53 µM; SI > 16.76), and the in vivo evaluation of this efficacy is demanded.^259^ Another type I interferon inducer, Polyinosinic:polycytidylic acid (poly(I:C)), is an analog of dsDNA with a powerful ability to reduce MERS‐CoV load in animal models.^192^ And phase II clinical trials demonstrated that it was well tolerated by malignant gliomas patients when injected intramuscularly.^322^, ^323^ Overall, the use of interferons or interferon inducers may be a valuable strategy against CoVs infection and should be furtherly accessed in animal models.

#### 4.3.2 Antiviral agents based on host entry factors

Several host factors are considered essential to CoVs entry, such as the host receptors that bind to CoVs S protein, and host proteases that facilitated CoVs entry through the cell surface or endosomal pathway. Thus, these factors become attractive targets for anti‐CoV agents’ development (Table 3). Antibodies, peptides, and some functional inhibitors targeting the host receptors can effectively interrupt the binding between S protein and the host cells. One example is that the N‐(2‐aminoethyl)‐1‐aziridine‐ethanamine (NAAE), a small molecular inhibitor, is able to target ACE2 leading to the block of S protein‐mediated membrane fusion, so does the synthetic peptides analogous, P4 and P5.^324^, ^325^ Similar agents were also found in MERS treatment, one of which, YS110, was confirmed to be well tolerated in patients with advance solid tumors.^326^ Owing to their specificity to appointed receptors, they were regarded as narrow‐spectrum drugs. However, the efficacy of these receptor‐targeted agents have never been evaluated in any CoVs‐infected patients and the safeties of these agents also remain unclear.

Host protease such as cathepsins and TMPRSS2 play a key role in the cleavage of S protein and the suppression of these proteases with potent inhibitors can obstruct the virus entry through either endosomal pathway or cell surface pathway. K11777 is a cathepsin inhibitor with broad spectrum against enveloped RNA virus including SARS‐CoV and MERS‐CoV.^237^, ^327^, ^328^ However, the camostat mesylate, applied in chronic pancreatitis treating, is a TMPRSS2 inhibitor that interrupts the TMPRSS2‐mediated cell surface entry.^329^, ^330^ The combination of cathepsin inhibitors and TMPRSS2 inhibitors is crucial to the complete suppression of MERS‐CoV in vitro. Inhibition of another host protease, furin, which is vital to furin‐mediated S cleavage for CoVs, also can block membrane fusion and the viral entry like MERS‐CoV.^143^ Notably, inhibition of host proteases may result in some side effects and need further evaluation.

Some approved antipsychotic agents (chlorpromazine, triflupromazine, and fluphenazine) and cardiotonic steroids (ouabain and bufalin) can inhibit clathrin‐mediated endocytosis via affecting the assembly of clathrin‐coated pits at the plasma membrane and binding sodium/potassium‐transporting ATPase subunit α1 (ATP1A1), respectively.^314^, ^331^, ^332^ Thus, they are considered as clathrin‐mediated endocytosis inhibitors. Alternatively, endosomal acidification also has a profound impact on endocytosis. Increase of endosomal pH shows an inhibitive effect on virus infection, which has been confirmed by chloroquine, a widely used autoimmune disease and antimalarial agents.^333^ Chloroquine proves to be active against a wide range of RNA viruses including HCoV‐229E, HCoV‐OC43, SARS‐CoV, and MERS‐CoV.^334^, ^335^, ^336^, ^337^, ^338^ Recently, chloroquine is identified to function at both entry and postentry phase of the SARS‐CoV‐2 infection with the EC90 value of 6.90 µM in Vero E6 cells.^259^ Additionally, as an immunoregulator, its antiviral activity may be synergistically intensified in vivo.^259^ Therefore, chloroquine is suggested as a potential option against the emerging SARS‐CoV‐2. Significantly, higher dose of chloroquine should not be recommended for severe COVID‐19 patients owing to the its drug safety, particularly while simultaneously accepting azithromycin and oseltamivir treatment, which was presented by a randomized clinical trial (NCT04323527).^339^ Hydroxychloroquine, a chloroquine analog with lower toxicity, was listed as a potential anti‐SARS‐CoV‐2 agent and recommended to be applied in COVID‐19 treatment by Chinese national guideline and FDA.^340^ However, evidence of the benefits and harms of hydroxychloroquine therapy is still insufficient and conflicting. Some small studies show that hydroxychloroquine was capable of decreasing SARS‐CoV‐2 shedding that could be enhanced by the combination of azithromycin and achieving a shorter time to clinical recovery.^341^, ^342^ But almost no clinical benefit was observed in other studies.^340^, ^343^ Therefore, therapeutic efficacy of hydroxychloroquine is still needed to be reconfirmed. Moreover, there are several factors required to be reconsidered before efficacy evaluation, such as the severity of illness, definition of the endpoints, and effects of the comorbidities.

#### 4.3.3 Other antiviral agents

Except for the innate immune response, host receptors, and other factors affecting the endocytosis, some signaling pathways have also been suggested as useful approaches for discovering anti‐CoV reagents (Table 3). Cyclophilins are peptidyl‐prolyl isomerases with physiological functions showing as modulating the calcineurin/NFAT pathway via reacting with CoVs nsp1, which is important for the immune cell activation.^244^ In addition, they are also required for the replication of numerous viruses including HIV, HCV, as well as CoVs.^344^ Consequently, inhibition of cyclophilins by cyclosporines, such as cyclosporin A (CsA) and its derivatives, has shown to block the replication of a wide range of CoVs.^244^, ^245^, ^345^, ^346^ However, the obvious immune suppressive properties of CsA limit its application in antiviral therapy. But alisporivir, a nonimmunosuppressive cyclosporin A‐analog, also displayed the inhibition to the CoVs replication at a low‐micromolar concentration.^347^ Additionally, the combined use of cyclosporine and interferon or ribavirin in vitro was beneficial to inhibit SARS‐CoV or MERS‐CoV infection, which needed to be furtherly evaluated in animal models.^347^, ^348^ Furthermore, some antiviral agents inhibiting the cellular signaling pathways, in particular, the ERK pathway and PI3K/AKT pathway, also interrupt the replication of CoVs.^243^, ^314^, ^349^ However, the efficacy and safety against CoVs infection of these agents still need to be reconsidered.

Corticosteroids, which were used in SARS and MERS treatment, have been linked to several complications such as psychosis, diabetes, and avascular necrosis.^350^, ^351^ They also were pointed out to be associated with viral replication prolongation in MERS patients.^351^ However, an update on the efficacy of dexamethasone based on a press release publicized recently indicated that severe COVID‐19 patients given 6 mg dexamethasone once daily shown a lower mortality (about 8‐26%) compared to patients with standard care.^352^, ^353^ Besides, another agent, methylprednisolone, also exhibited potential capacity in reducing the mortality rate and achieving better clinical outcomes in severe COVID‐19 patients.^354^, ^355^ Thus, it is wise to apply corticosteroids to the right patients at the right time. But physicians also need to monitor the corticosteroid‐related complications. Clinical trials of corticosteroid treatments are shown in Table 3.

### 4.4 Potential immunotherapeutic options

#### 4.4.1 Antibody and plasma therapy

S protein of SARS‐CoV proves to be highly immunogenic during infection and responsible for eliciting a humoral immune response in the host.^356^ Antivirus antibodies could be detected in the plasma of convalescent patients’ infected SARS‐CoV and MERS‐CoV.^357^, ^358^ Convalescent plasma therapy has been applicated in treating patients infected by numerous viruses involving Ebola virus, Junin virus, Machupo virus, and Lassa fever.^359^, ^360^, ^361^, ^362^ As for SARS‐CoV, higher day‐22 discharge rate and lower mortality rate have been observed among SARS patients who received convalescent plasma transfusion before day 14 of the illness.^363^, ^364^ This is consistent with another small cohorts research concluding infected patients with severe conditions who failed to respond to current therapies but finally survived after transfused with convalescent plasma, with no obvious side effects.^358^ Similar results were found in MERS patients.^365^ Additionally, plasma adoptive therapy with anti‐MERS‐CoV antibodies could block the virus adhesion and accelerate the viral elimination from MERS‐CoV–infected animal models.^192^ But the efficacy and safety of convalescent plasma therapy in COVID‐19 patients still need to be reevaluated. Although convalescent plasma therapy proves to be a potentially effective therapeutic option for emerging CoVs, several factors still limit its extensive use in clinical, one of which is enough titers of serum neutralizing antibodies.

The development of mAbs targeting the S protein of CoVs is regarded as a remedial strategy (Table 4). Potent mAbs against S protein of human CoVs can be attained via transgenic mice immunization, convalescent B cells immortalization, and cloning of small chain variable regions from naïve and convalescent patients.^366^ The majority of mAbs interact with the RBD of S protein leading to the interruption of RBD‐receptor binding and block the viral attachment. A few mAbs react with other regions of S protein besides the RBD.^366^ Although binding to different epitopes, these mAbs exhibit capacity to reduce the viral titers. Two RBD‐specific neutralizing mAbs, MERS‐4 and MERS‐27, which were derived from single‐chain variable regions, revealed suppressive effects against both MERS‐CoV and pseudotyped MERS‐CoV infection at nanomolar concentrations and were recommended as promising candidates for therapeutic interventions to MERS.^232^ Based on similar mechanisms, other human mAbs for MERS‐CoV were also capable of competing with DPP4 for RBD binding and neutralizing the virus.^233^, ^234^, ^367^, ^368^, ^369^, ^370^ When administrated to individuals at risk, some of the mAbs were capable of preventing viral replication and contributing to block the transmission of MERS‐CoV among human.^368^ Thus, such antibodies could be served as prophylactic strategies in clinical and valuable tools to guild the development of effective anti‐CoVs vaccines.^234^, ^368^

**TABLE 4:** Potential immunotherapeutic options for HCoVS

Therapeutic options	Design	Antiviral spectrum	Status	Refs.
**Convalescent plasma**	Plasma from convalescent patients infected with viruses	Broad‐spectrum SARS‐CoV MERS‐CoV SARS‐CoV‐2	Clinical trials	^346^, ^351^, ^352^, ^353^
**MERS‐4, MERS‐27, m336, m337, m338, REGN3051 and REGN3048**	Targeting S protein and attaining via transgenic mice immunization, convalescent B cells immortalization, and cloning of small chain variable regions from naïve and convalescent patients	SARS‐CoV MERS‐CoV	Preclinical Phase I clinical trial for MERS	^231^, ^232^, ^233^, ^361^, ^362^, ^363^, ^364^
**Inactivated virus vaccines**	Rendering the viral genome noninfectious with chemicals or radiation while maintaining the virion structure	SARS‐CoV MERS‐CoV SARS‐CoV‐2	Preclinical Phase I/ II clinical trial for COVID‐19	^365^, ^366^
**live‐attenuated virus vaccines**	Reducing or eliminating the virulence of a live virus with chemical‐driven or site‐directed mutagenesis	SARS‐CoV MERS‐CoV	Preclinical	^365^, ^366^
**Viral vector vaccines**	Viral vectors (MVA or Ad) that expressing full‑length or the S1 subunit of S protein	SARS‐CoV MERS‐CoV SARS‐CoV‐2	Preclinical Phase I/ II clinical trial for COVID‐19 and MERS	^365^, ^366^
**Nanoparticles**	Purified S protein‑containing nanoparticles produced in insect cells, which were infected with specific recombinant baculovirus containing the gene encoding S protein of CoVs	SARS‐CoV MERS‐CoV SARS‐CoV‐2	Preclinical Phase I clinical trial for COVID‐19	^386^
**Recombinant protein subunits vaccines**	Antigenic components either full‐length S protein or RBD subunit of S protein of CoVs	SARS‐CoV MERS‐CoV SARS‐CoV‐2	Preclinical Phase I clinical trial for COVID‐19	^365^, ^366^
**DNA vaccines**	DNA encoding viral antigenic components	SARS‐CoV MERS‐CoV SARS‐CoV‐2	Preclinical Phase I clinical trial for SARS Phase I/ II clinical trial for COVID‐19 and MERS	^365^, ^366^

Abbreviations: CoV, coronavirus; MERS, Middle East respiratory syndrome; SARS, severe acute respiratory syndrome.

#### 4.4.2 Vaccine

From SARS‐CoV to SARS‐CoV‐2, the emergence of severe human CoVs have taught us many lessons about the importance of rapid diagnostics and effective vaccines to control the outbreak caused by these viruses. Due to the persistence of zoonotic sources in endemic areas, lethal CoVs remain existing in human society and may lead to the epidemic at any time. Thus, a priority is to develop vaccines targeting conserved alleles and providing broad‐spectrum protection against varied viral strains. Since the emergence of SARS‐CoV and MERS‐CoV, several strategies were applicated in vaccine design, including inactivated virus vaccines, live‐attenuated virus vaccines, viral vector vaccines, nanoparticles, recombinant protein subunits vaccines, and DNA vaccines (Table 4).^371^, ^372^ And clinical trials have also been developed to test the efficacy of the novel vaccines (Table 5). Effective vaccines are pivotal in blocking the virus spread from animals’ reservoirs to human hosts. Inactivated virus vaccines, preserving the viral structure and antigenicity but eliminating the infectious ability, could elicit neutralizing antibodies in animal models of SARS‐CoV and show protection against viral replication when administrated with or without adjuvants.^372^, ^373^, ^374^, ^375^ Different from inactivated virus vaccines, live attenuated virus vaccines are generated via reducing the virulence of live viruses, meaning that they are still able to induce infection, which may be related to the disseminated infection observed in immunocompromised patients. In addition, live‐attenuated virus vaccines can induce an innate and adaptive immune response and the protective value can last for a long time.^371^ Besides, other strategies for vaccines development are also evaluated in animal models. Based on the experience of SARS‐CoV and MERS‐CoV, the development of novel SARS‐CoV‐2 vaccine is currently underway and requires more research.

**TABLE 5:** Important clinical trials with vaccines for SARS‐CoV, MERS‐CoV, and SARS‐CoV‐2

HCoVs	Trial	Phase	Status	Sample size	Types	Design
**SARS‐CoV**	NCT00099463	Phase I	Completed	10	DNA vaccine	A recombinant DNA vaccine, VRC‐SRSDNA015‐00‐VP.
**SARS‐CoV‐2**	NCT04299724	Phase I	Recruiting	100	Viral vector vaccine	Pathogen‐specific aAPC
	NCT04276896	Phase I/ II	Recruiting	100	Viral vector vaccine	LV‐SMENP DC and antigen‐specific cytotoxic T cell vaccines
	NCT04283461	Phase I	Recruiting	45	Nanoparticles mRNA‐based vaccine	mRNA‐1273
	NCT04352608	Phase I/ II	Recruiting	744	Inactivated vaccine	SARS‐CoV‐2‐inactivated vaccine
	NCT04412538	Phase I/ II	Recruiting	942	Inactivated vaccine	SARS‐CoV‐2‐inactivated vaccine
	NCT04405908	Phase I	Recruiting	150	Recombinant S protein subunits vaccines	A recombinant SARS‐CoV‐2 trimeric S protein subunit vaccine, SCB 2019
	NCT04445389	Phase I/ II	Recruiting	190	DNA vaccine	A DNA vaccine, GX‐19
	NCT04368728	Phase I/ II	Recruiting	7600	RNA vaccine	RNA vaccine, BNT162a1, BNT162b1, BNT162b2, BNT162c1
	NCT04368988	Phase I	Recruiting	131	Nanoparticle vaccine	A SARS‐CoV‐2 recombinant spike protein nanoparticle vaccine (SARS‐CoV‐2 rS) with Or without MATRIX‐M™ adjuvant
	NCT04453852	Phase I	Recruiting	40	Recombinant protein subunits vaccines	Recombinant protein SARS‐COV‐2 vaccine, Covax‐19
**MERS‐CoV**	NCT03399578	Phase I	Recruiting	48	Viral vector vaccine	ChAdOx1 MERS
	NCT03615911	Phase I	Completed	26	Viral vector vaccine	MVA‐MERS‐S
	NCT04170829	Phase I	Recruiting	24	Viral vector vaccine	ChAdOx1 MERS
	NCT04130594	Phase I/ II	Recruiting	162	Viral vector vaccine	BVRS‐GamVac
	NCT04128059	Phase I/ II	Recruiting	268	Viral vector vaccine	BVRS‐GamVac‐Combi
	NCT04119440	Phase I	Not yet recruiting	160	Viral vector vaccine	MVA‐MERS‐S_DF1
	NCT02788188	Phase I	Completed	38	mAbs	SAB‐301
	NCT03721718	Phase I/ II	Active, not recruiting	60	DNA vaccine	GLS‐5300
	NCT02670187	Phase I	Completed	75	DNA vaccine	GLS‐5300

However, several concerns should be addressed about the vaccination. The first is the disease deterioration caused by vaccination. Although this situation only appears in a small subset of SARS vaccine studies, it is still a significant problem that needs to be properly solved.^372^ Second, the variability of S protein can mediate CoVs escape from neutralization, suggesting that recombinant protein subunits vaccines based on S protein may demand multivalent approaches.^376^ Last but not least, how to define the target individuals suitable for the vaccination. It is recommended that people at a high risk of CoV exposure such as health care workers should be vaccinated.^377^

## 5 CONCLUSION AND OUTLOOK

The emergence and prevalence of highly pathogenic CoV severely threaten public health. A task of top priority is to make clear the viral structural and epidemiological characteristics and block the viral dissemination as well as the progression of the disease, at the first case. To date, further understanding of the life cycle and the pathogenesis of emerging human CoVs makes current therapeutic strategies of antiviral infection more rational. Repurposing existing antiviral drugs is an effective short‐term strategy to deal with emerging CoV such as the ongoing SARS‐CoV‐2. Various agents with different targets have been evaluated in vitro and in vivo. But not all antiviral agents are capable of achieving better efficacy than in vitro, and in vivo studies are needed to select optimal agents. Suitable animal models are particularly significant. However, there are only a few effective animal models available for the studies of CoVs treatment, which may postpone the clinical evaluation of drugs. Besides, due to the diversity of viruses and the capacity of rapidly mutating, some antiviral reagents available for existing CoVs may become invalid. Accordingly, sequencing more natural specimens and combination therapy with two or more synergistic agents have become promising solutions. Furthermore, the efficacy of current antiviral therapies needs to be estimated in larger scale, well‐organized randomized clinical trial

The efficacy and safety of the most practicable strategies for the ongoing COVID‐19 are urgently needed to be further assessed in clinical trials, involving remdesivir (GS‐5734), lopinavir/ritonavir, lopinavir/ritonavir combined with IFN‐β, convalescent plasma, and mAbs, which prove to be potentially effective options against SARS‐CoV‐2. Additionally, to control the future CoV pandemics, the development of novel broad‐spectrum antiviral agents covering numerous CoVs will become the ultimate goal.

## CONFLICT OF INTEREST

The authors declare no conflict of interest.
